# Pattern and management of maxillofacial fractures in Jordanian children and adolescents

**DOI:** 10.4317/medoral.25712

**Published:** 2022-12-24

**Authors:** Anwar B Bataineh

**Affiliations:** 1 Professor of Oral and Maxillofacial Surgery, Faculty of Dentistry, Jordan University of Science and Technology, Irbid, Jordan

## Abstract

**Background:**

The aim of this study was to analyze the characteristics, etiology and treatment of maxillofacial fractures among children and adolescents in northern part of Jordan.

**Material and Methods:**

A retrospective cohort study which included 91 children and adolescents patients who were treated for maxillofacial fractures during a period of three years between January 2019 and December 2021 at a tertiary hospital in Jordan.

**Results:**

Over a period of three years, a total of 91 children between the age of 0 and 19 years were treated with 156 total maxillofacial fractures. Of these, 68 (74.73%) were males and 23 (25.27%) were females. One tenth of patients (10 (10.99%) were children of the preschool group and 55 patients (60.44%) were adolescents. Road traffic accident (RTA) was the most common cause of maxillofacial fractures, accounting for 57 (62.64%) of cases. Mandibular fractures were the most common and accounted for 82 (90.2%) of all fractures, followed by the zygomatic bone fractures 40 (44%). The most common treatment was intermaxillary fixation (IMF) with 53 (33.97%) fractures.

**Conclusions:**

Maxillofacial fractures are predominant among adolescents in comparison to children. RTA was the most common cause of maxillofacial fractures, mandibular fractures were the most common fractures, and intermaxillary fixation (IMF) was the most common treatment modality.

** Key words:**Trauma, mandible, zygomatic complex, maxilla, treatment.

## Introduction

Children and adolescents are distinctive individuals according to their age and the stage of their bone maturation. In relation to injury, their characteristics of oral and maxillofacial fractures are different (1), but injury is much less common in children than in adults, due to the parental supervision (2). Etiology and patterns of children and adolescents’ maxillofacial fractures differs from adult because of anatomical factors and growth and vary depending on the levels of socioeconomic, cultural and environmental factors. Fall is a common cause of injury in children while road traffic accident (RTA), sports and violence are common in adolescents. Maxillofacial fractures in children and adolescents account for less than 15% of all facial fractures (3), despite that, maxillofacial fractures remain one of the most common causes of morbidity and mortality in children and adolescents (4). Previous studies found that maxillofacial injuries are rare before the age of 5 years, with their incidence progressively increasing from the beginning of school to adolescence (5).

Management of children and adolescent’s maxillofacial fractures must be planned by taking into consideration that children and adolescents are in the growth and development stage. Understanding the characteristics of maxillofacial fractures in children and adolescents can help in performing accurate diagnosis and appropriate treatment methods (6). Poor resource management can lead to numerous complications, such as growth disturbances and temporomandibular joint ankylosis (7,8). The management of maxillofacial fractures in children and adolescents in developing countries have challenges, these include personnel training programs that focus on enhancing management skills for clinical staff and lack of public awareness.

The aim of this study was to analyze the characteristics, etiology and treatment of maxillofacial fractures among children and adolescents in northern part of Jordan.

Material and Mehods

A retrospective cohort study included 91 children and adolescents patients who were treated for maxillofacial fractures during a period of three years between January 2019 and December 2021at the King Abdullah University Hospital/Jordan University of Science and Technology, Irbid, Jordan. The data were collected from the medical records of all children and adolescents. This ethical approval was waived by the Institutional Ethical Review Committee of the university due to the retrospective nature of this study, and was conducted according to the Declaration of Helsinki.

The study collected data on patients aged from 0 to 19 years old with maxillofacial fractures who had been treated. The inclusion criteria were patients who had maxillofacial fractures whether admitted to the hospital or treated as outpatients. The exclusion criteria was patients aged over 19 years, incomplete information in their medical record or having a history of previous or pathological maxillofacial fractures.

The data of age, gender, causes (fall, road traffic accidents, violence, sports injury, gunshot), anatomic site, pattern of maxillofacial fracture, and treatment methods were recorded and analyzed. Age was categorized into preschool age [0-6 years], school age [7-12 years], and adolescent [13-19 years]. Maxillofacial fractures were classified as mandibular, maxillary, and zygomatic. Plain radiographs and computed tomography (CT) scans were used for diagnosis.

Mandibular fractures were classified as dentoalveolar, symphysis, parasymphysis, body, angle, ramus, and condyle fractures. Maxillary fractures were classified as dentoalveolar, LeFort I, LeFort II, LeFort III, and maxillary sinus. Zygoma fractures were categorized as zygomatic arch zygomatic complex, zygomaticomaxillary suture (ZMS), and zygomaticofrontal suture (ZFS) fractures. On the basis of examination and investigations, a suiTable treatment approach was carried out. Treatment was planned to be noninvasive and whenever possible conservative and to prevent growth disturbance, minimal manipulation was used. Treatment was classified as conservative, closed reduction, or open reduction with internal fixation plates or wires [ORIF (Plates) or (Wires)], Intermaxillary fixation (IMF). Ages 0 to 6: conservative treatment; ages 7 to 12: intermaxillary fixation (IMF) maintained for 3 to 4 weeks. Children with Greenstick fractures should receive observation and follow-up every two to three days for two to four weeks, and patients aged 13 to 19 should undergo either a closed or an open reduction depending on the severity of the fracture. The preferred method of fixation will depend on the patient's age and anatomic site.

Data were analyzed using SPSS, Version 27. Categorical data were presented as frequency and percentages. A Chi-Square test was performed to compare proportions. A value of p ≤0.05 was considered statistically significant.

## Results

Over a period of three years, a total of 91 children between the ages of 0 and 19 years were treated with 156 maxillofacial fractures. Of these, 68 (74.73%) were males and 23 (25.27%) were females with a Male:Female ratio of 2.96:1. There was an increase in the number of cases as the age advanced. One tenth of patients (10 (10.99%) were children of the preschool group, 55 patients (60.44%) were adolescents. The mean age of the patients was 13.88 years (Table 1).

RTA was the most common cause of maxillofacial fractures, accounting for 57 (62.64%) of cases. The second most common cause was fall and violence accounting for 15 (16.48%) for each (Table 2).

The distribution of maxillofacial fractures according to anatomic site revealed that mandibular fractures were the most common and accounted for 82 (90.2%) of all fractures, followed by the zygomatic bone fractures 40 (44%). The most common site of mandibular fractures was angle 22 (26.83%), followed by parasymphysis 18 (21.95%). The lowest affected site was the mandibular condyle 3 (3.66%). The most common site of maxillary fractures was Le Fort I which accounted for 12 (35.30%). Zygomatic arch was the most common site accounting for 20 (50%) of zygomatic bone fractures, as shown in Table 3.

**Table 1:**
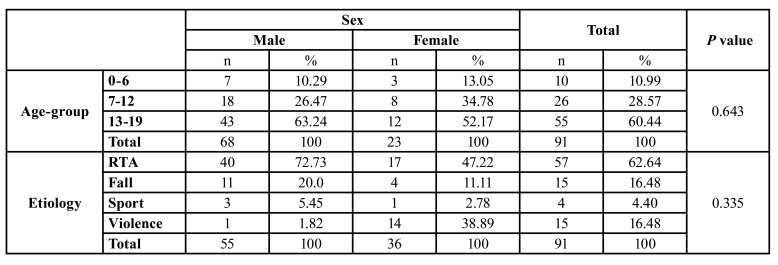
Distribution of age and etiology of maxillofacial fractures in children and adolescents according to Sex.

**Table 2:**
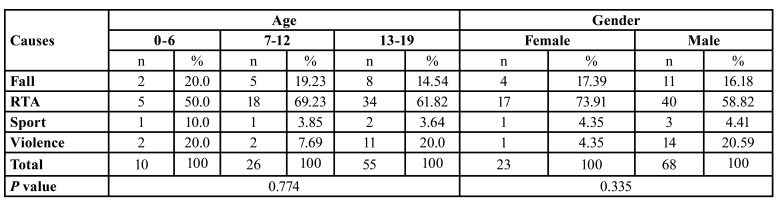
Distribution of etiology according to age and sex.

**Table 3:**
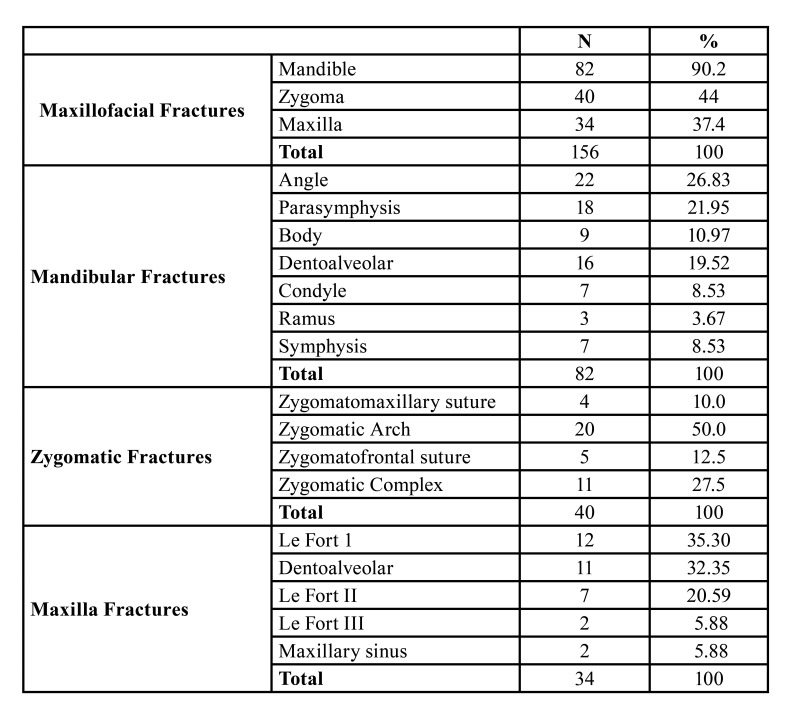
Distribution of maxillofacial fractures according to anatomic site.

The most common treatment was intermaxillary fixation (IMF) with 53 (33.97%) fractures, while ORIF (Plates) was in 30 (19.23%) fractures. The most frequently affected site of the mandibular fractures was the angle 22 (14.10%) fractures, maxillary fractures was Le Fort I 12 (7.69%) and of the zygomatic fractures was zygomatic arch 20 (12.82%) fractures (Table 4). Associated injuries were significantly more common in dentoalveolar (19.5% of patients) and condylar fractures (8.5% of patients) than from other sites of fractures (*p* < 0.001).

**Table 4:**
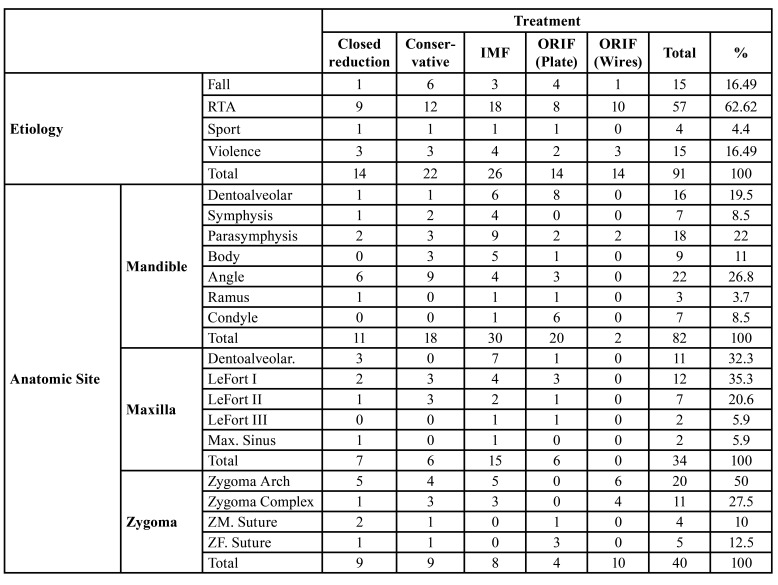
Distribution of treatment of maxillofacial fractures in children and adolescents according to etiology and anatomic site.

## Discussion

Children and adolescents maxillofacial fractures had different etiologies, and their incidence varied according to patients’ age, gender and account for less than 15% of the total number of maxillofacial fractures (9). The children and adolescents patients were distributed in three distinct age groups; 0-6 years, 7-12 years and 13-19 years, respectively. This distribution was preferred because it comprises three important periods in the development of children and adolescents patients, namely preschool, school and teenagers (adolescents).

In line with previous studies, this study found that maxillofacial fractures are rare before the age of 5 years, with their incidence progressively increasing from the beginning of school to adolescence (5,10), and the mean age of the patients was 13.88 years.

The results of this study demonstrated an increase in the number of cases as the age advanced with a high frequency found in the adolescent group which consisted of 55 patients (60.44%), comprising 43 males and 12 females. Similar findings were also presented in many studies in the world (9,11,12). On the contrary, other studies found the primary school age group to have the highest prevalence among other groups (13-15).

In this research, maxillofacial fractures in children and adolescents accounted for 14.8% of all fractures diagnosed for patients treated. The results are in line with the results of other studies, which reported an incidence of 14.7% (3), 14.6% (16), all of which were below the 15% threshold. A single study carried out in India reported an incidence of only 1.01% of maxillofacial fractures in the children and adolescents population under the age of 16 years (17), which was well below the incidence reported in the present study. While our peak age-group was reported to be 16 to 19 years, other studies have reported lower peak age groups (18,19).

Studies have shown a higher incidence rate of children and adolescents maxillofacial fractures in males, with male-to-female ratios ranging from 1.5:1 to 2.8:1, in this study, the male-to-female ratio was 2.96:1, which was in line with several recent studies and previously published work (5,20). Another study identified a male to female ratio of 16.6:1 which is extremely high (12).

In this study, RTA was the most common cause of maxillofacial fractures in children and adolescents, accounting for 57 (62.64%) of cases among all age groups. This is consistent with other studies (12,17,20) where the main cause of maxillofacial fractures in children and adolescents was RTA. Other studies demonstrated that falls were the most frequent cause of maxillofacial fractures in children and adolescents (20,21), while other studies revealed that the main etiological factor in the occurrence of maxillofacial fractures was interpersonal violence. In the present study, fall and violence which accounted for 15 (16.48%) each were the second most common cause of maxillofacial fractures in our patient population, which is in line with other studies (5,16).

It was shown in a recent study that mandibular fractures were the most common which accounted for 82 (90.2%) of all maxillofacial fractures in children and adolescents, followed by the zygomatic bone fractures 40 (44%), maxillary fractures 34 (37.4%), which is in line with other studies (21). In the present study, the most common site of mandibular fractures was angle 22 (26.83%) and the lowest affected site was the mandibular condyle 3 (3.66%).

The treatment of facial fractures in children and adolescents differs from the treatment of fractures in adults, considering the fact that children and adolescents patients have active growth and development (22). In the literature, conservative treatment of facial fractures ranged from 82% reported by Eggensperger Wymann *et al*. (23) and 21% reported by Ferreira *et al*. (24). For most of non-displaced mandibular fracture especially the condylar fractures, it was recommended to perform the closed reduction internal fixation based on occlusion restoration with or without IMF followed by physiotherapy.

Moreover, due to problems associated with interference with the growth and development of the facial skeleton, as well as with normal dental development, a minimalist approach, such as no treatment or closed reduction is often the preferred management for maxillofacial fractures, especially of the mandibular condyle, in preschool and school age children (10,16,25,26). On the contrary, as patients grow older, the craniofacial skeleton approaches adult maturity, and ORIF is performed more frequently (16,25,26). This was confirmed in this study, as only 7% of preschool children underwent ORIF, but this increased to almost 50% in the adolescent group, indicating a significant correlation with age.

Few of the cases were managed by observation, medications, and soft diet in the under 5-year patients. The maxillary fractures were treated with IMF and elastic traction if the teeth were adequately erupted; if not then ORIF was used.

In the recent study, the most common treatment was intermaxillary fixation (IMF) with 53 (33.97%) fractures, while ORIF (Plates) was in 30 (19.23%) fractures. This was a lower number when compared to other studies carried out by Motamedi (27) (40% ORIF cases) and Vetter *et al*. (28) (60% ORIF cases). The most frequently affected site of the mandibular fractures was the angle 22 (14.10%) fractures, maxillary fractures was Le Fort I 12 (7.69%) and of the zygomatic fractures was zygomatic arch 20 (12.82%) fractures. Associated injuries were significantly more common in dentoalveolar (19.5% of patients) and condylar fractures (8.5% of patients) than from other sites of fractures (*p* < 0.001). Similar treatment rate by ORIF in older pediatric age-group has been reported by Vetter *et al*. (28).

## Conclusions

Maxillofacial fractures are predominant among adolescents compared to children. RTA was the most common cause of maxillofacial fractures, mandibular fractures were the most common fractures, and intermaxillary fixation (IMF) was the most common treatment modality. The expansion of driving privileges over adolescence will lead to a rise in maxillofacial fractures in teenagers and children. To reduce the prevalence of maxillofacial fractures in children and teenagers, effective initiatives should be created as well as strategies to increase parental awareness. Further studies for the purpose of developing the most effective treatment, additional research with longer follow-up periods is advised.
